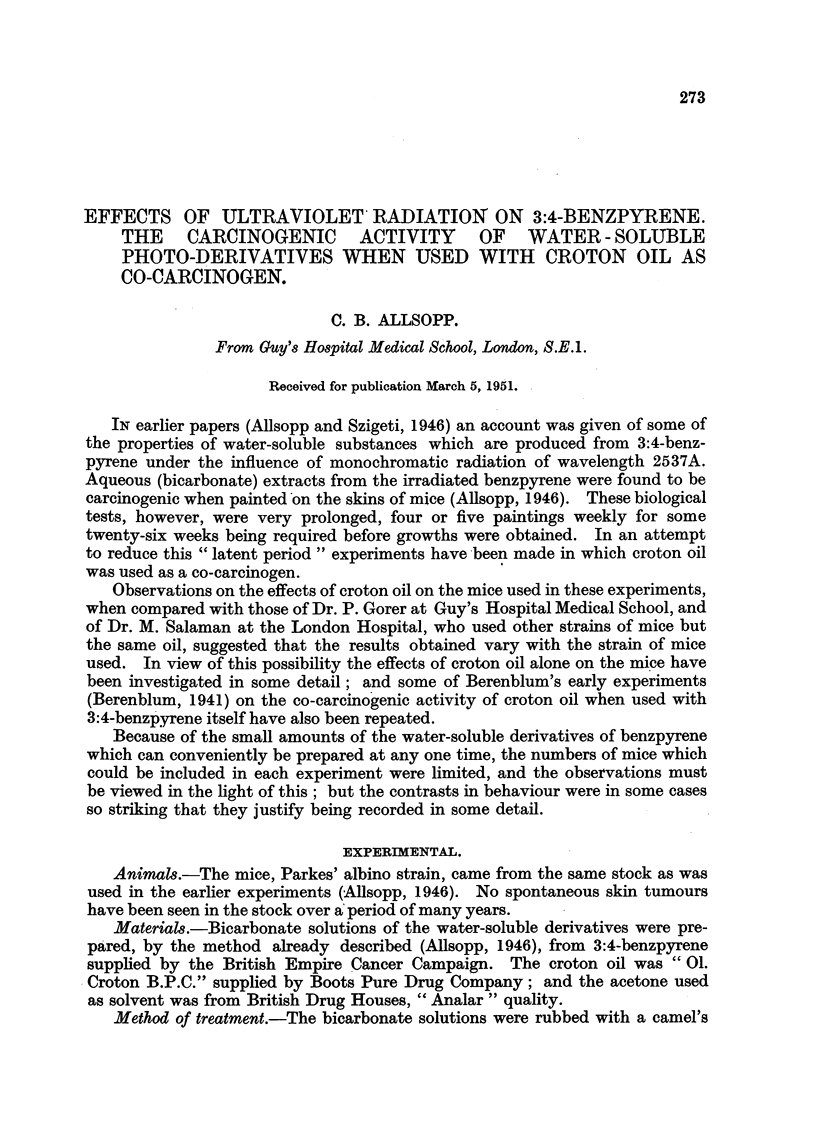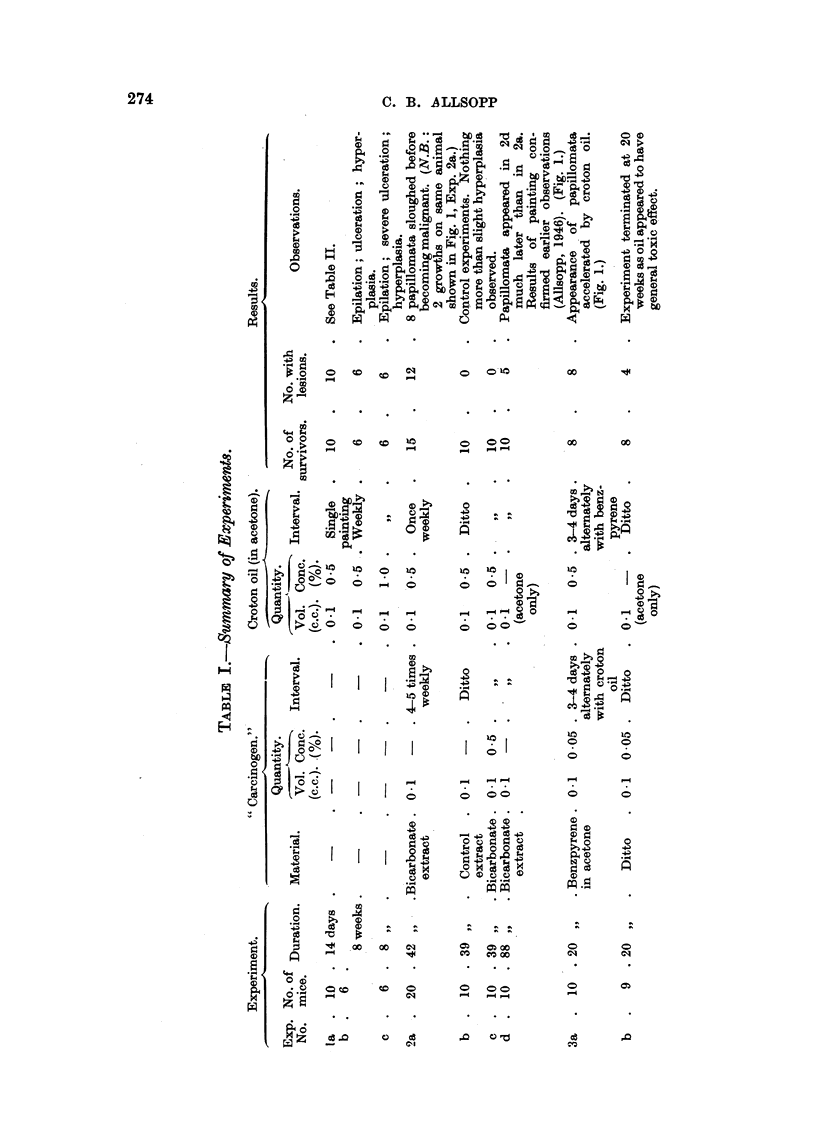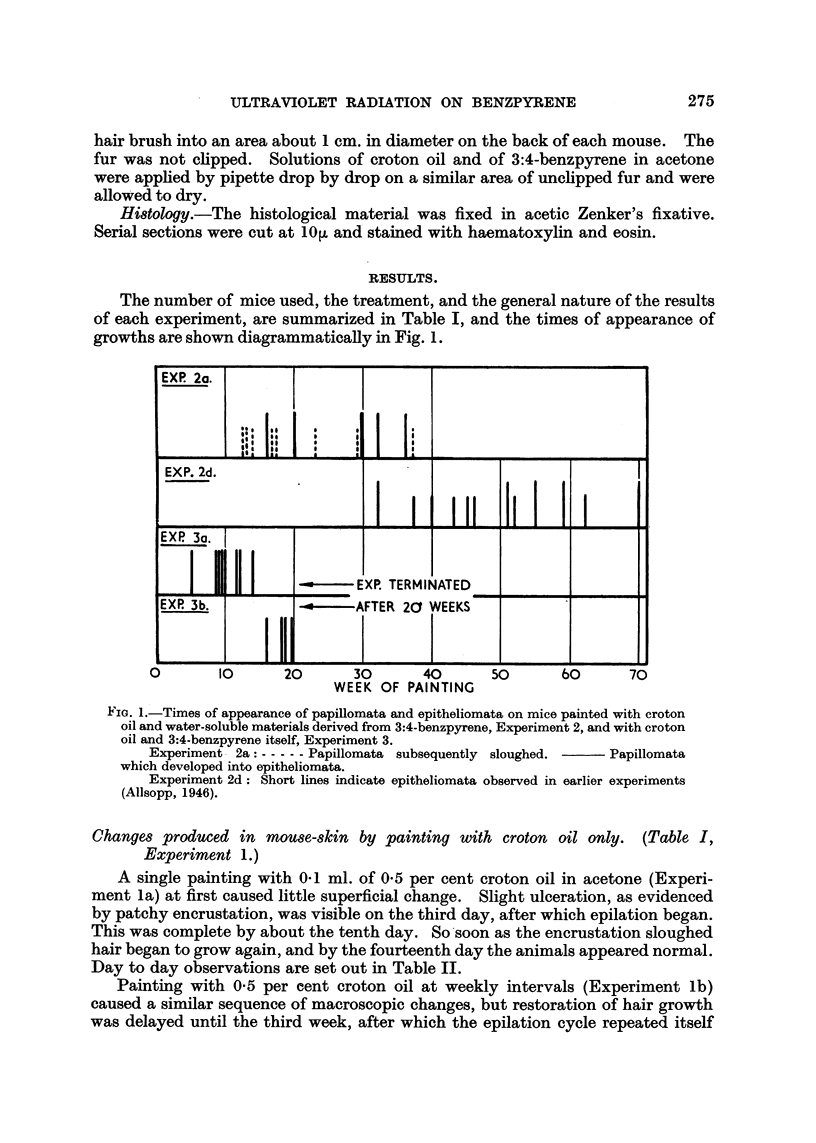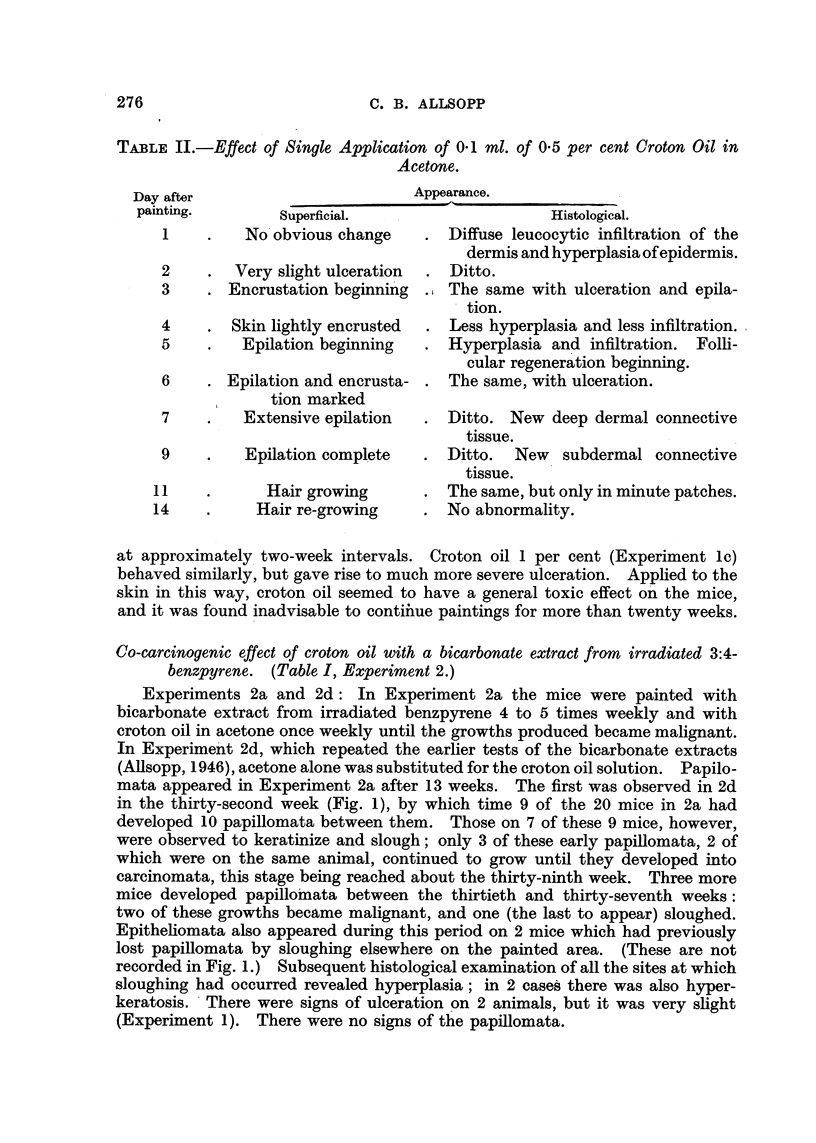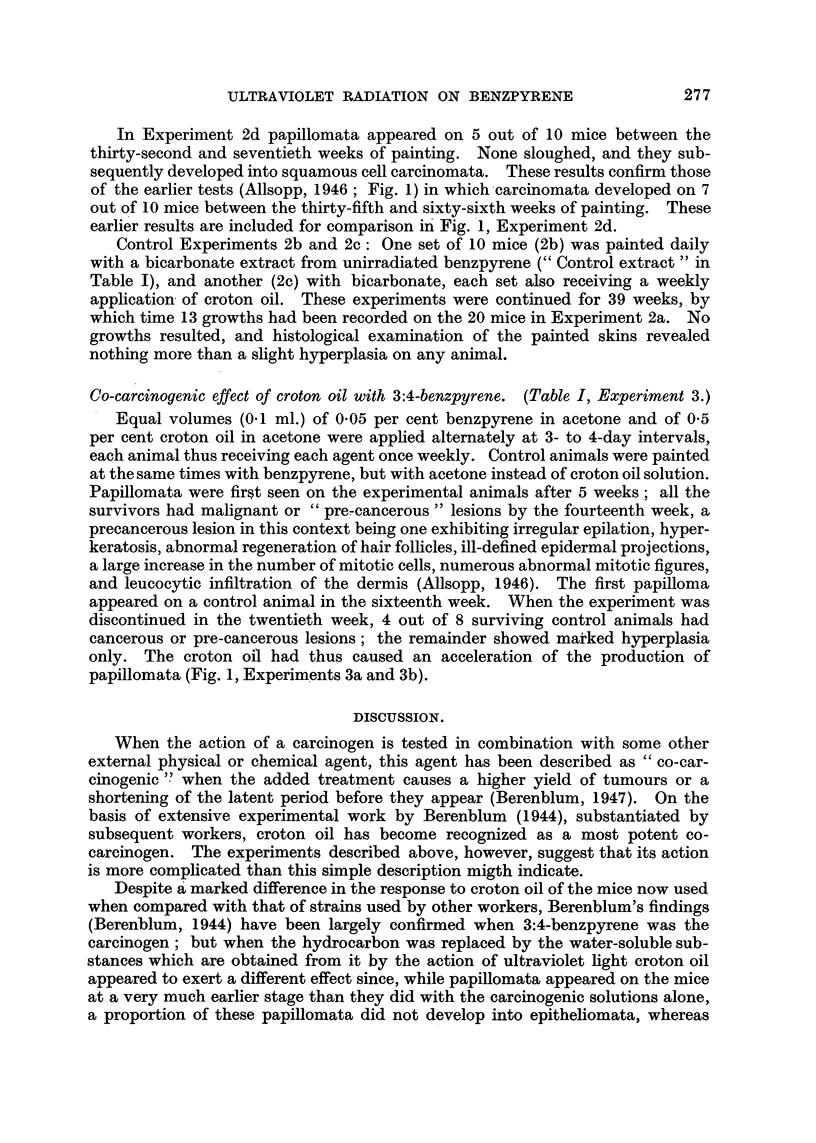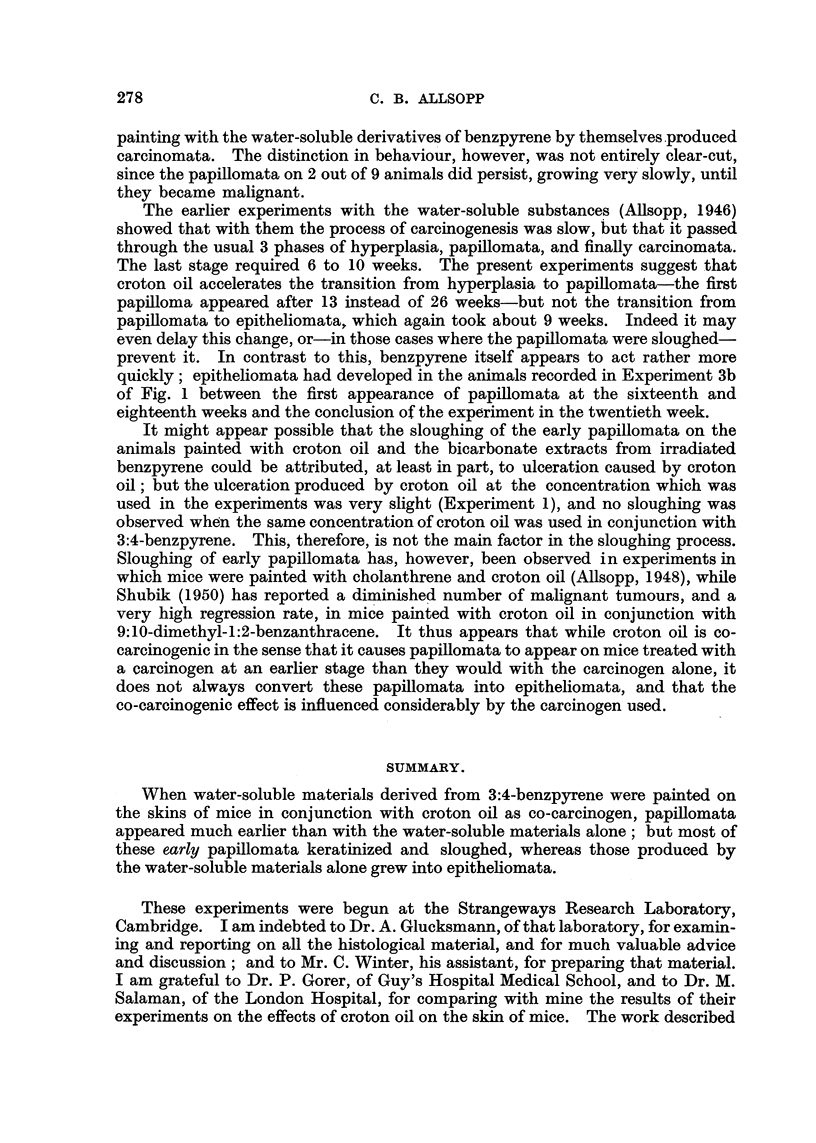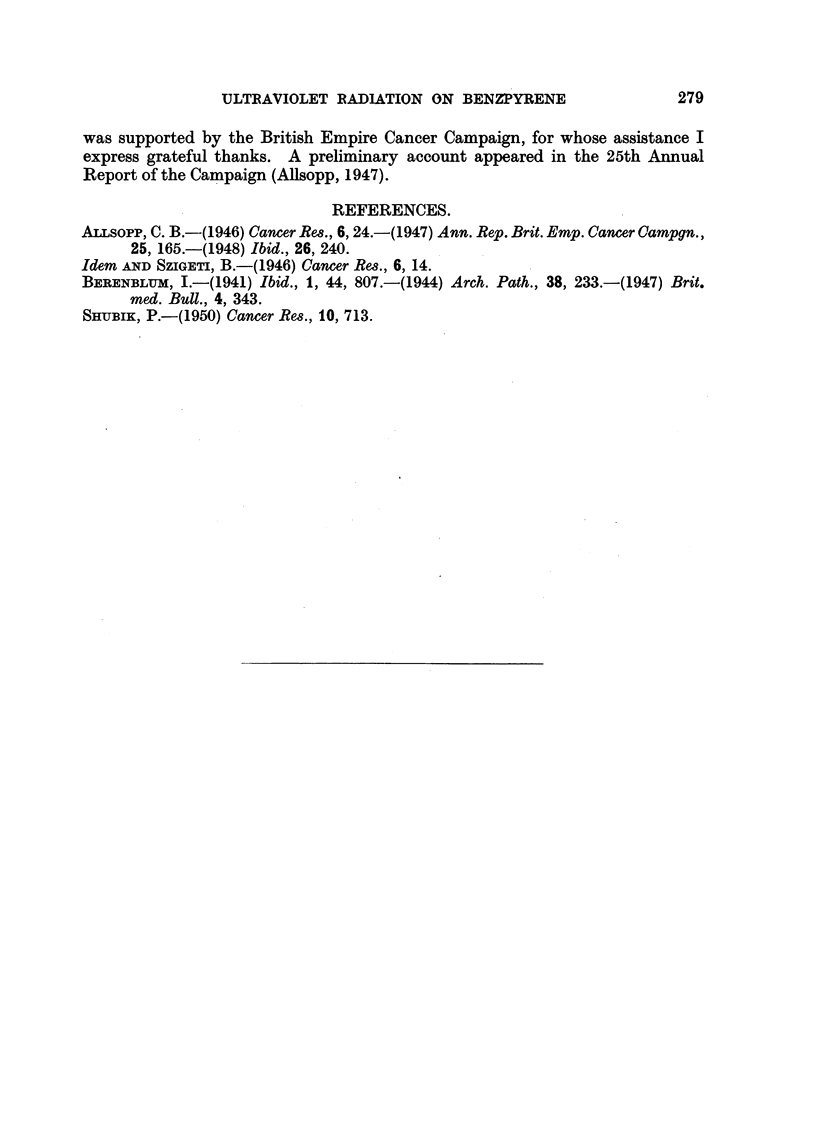# Effects of Ultraviolet Radiation on 3: 4-Benzpyrene. The Carcinogenic Activity of Water-Soluble Photo-Derivatives when used with Croton Oil as Co-Carcinogen

**DOI:** 10.1038/bjc.1951.28

**Published:** 1951-06

**Authors:** C. B. Allsopp


### EFFECTS OF ULTRAVIOLET RADIATION ON 3:4-BENZPYRENE. THE CARCINOGENIC ACTIVITY OF WATER-SOLUBLE PHOTO-DERIVATIVES WHEN USED WITH CROTON OIL AS CO-CARCINOGEN.

C. B. ALLSOPP.

*From Guy's Hospital Medical School, London, S.E.1.*

Received for publication March 5, 1951.

In earlier papers (Allsopp and Szigeti, 1946) an account was given of some of the properties of water-soluble substances which are produced from 3:4-benzpyrene under the influence of monochromatic radiation of wavelength 2537A. Aqueous (bicarbonate) extracts from the irradiated benzpyrene were found to be carcinogenic when painted on the skins of mice (Allsopp, 1946). These biological tests, however, were very prolonged, four or five paintings weekly for some twenty-six weeks being required before growths were obtained. In an attempt to reduce this " latent period " experiments have been made in which croton oil was used as a co-carcinogen.

Observations on the effects of croton oil on the mice used in these experiments, when compared with those of Dr. P. Gorer at Guy's Hospital Medical School, and of Dr. M. Salaman at the London Hospital, who used other strains of mice but the same oil, suggested that the results obtained vary with the strain of mice used. In view of this possibility the effects of croton oil alone on the mice have been investigated in some detail; and some of Berenblum's early experiments (Berenblum, 1941) on the co-carcinogenic activity of croton oil when used with 3:4-benzpyrene itself have also been repeated.

Because of the small amounts of the water-soluble derivatives of benzpyrene which can conveniently be prepared at any one time, the numbers of mice which could be included in each experiment were limited, and the observations must be viewed in the light of this; but the contrasts in behaviour were in some cases so striking that they justify being recorded in some detail.

### EXPERIMENTAL.

*Animals.*—The mice, Parkes' albino strain, came from the same stock as was used in the earlier experiments (Allsopp, 1946). No spontaneous skin tumours have been seen in the stock over a period of many years.

*Materials.*—Bicarbonate solutions of the water-soluble derivatives were prepared, by the method already described (Allsopp, 1946), from 3:4-benzpyrene supplied by the British Empire Cancer Campaign. The croton oil was " Ol. Croton B.P.C." supplied by Boots Pure Drug Company; and the acetone used as solvent was from British Drug Houses, " Analar " quality.

*Method of treatment.*—The bicarbonate solutions were rubbed with a camel's

TABLE I.—*Summary of Experiments.*

| Experiment. | | | "Carcinogen." | | | | Croton oil (in acetone). | | | Results. | | |
|---|---|---|---|---|---|---|---|---|---|---|---|---|
| Exp. No. | No. of mice. | Duration. | Material. | Quantity. Vol. (c.c.). | Quantity. Conc. (%). | Interval. | Quantity. Vol. (c.c.). | Quantity. Conc. (%). | Interval. | No. of survivors. | No. with lesions. | Observations. |
| 1a | 10 | 14 days | — | — | — | — | 0·1 | 0·5 | Single painting | 10 | 10 | See Table II. |
| b | 6 | 8 weeks | — | — | — | — | 0·1 | 0·5 | Weekly | 6 | 6 | Epilation ; ulceration ; hyperplasia. |
| c | 6 | 8 ,, | — | — | — | — | 0·1 | 1·0 | ,, | 6 | 6 | Epilation ; severe ulceration ; hyperplasia. |
| 2a | 20 | 42 ,, | Bicarbonate extract | 0·1 | — | 4–5 times weekly | 0·1 | 0·5 | Once weekly | 15 | 12 | 8 papillomata sloughed before becoming malignant. (*N.B.*: 2 growths on same animal shown in Fig. 1, Exp. 2a.) |
| b | 10 | 39 ,, | Control extract | 0·1 | — | Ditto | 0·1 | 0·5 | Ditto | 10 | 0 | Control experiments. Nothing more than slight hyperplasia observed. |
| c | 10 | 39 ,, | Bicarbonate | 0·1 | 0·5 | ,, | 0·1 | 0·5 | ,, | 10 | 0 | |
| d | 10 | 88 ,, | Bicarbonate extract | 0·1 | — | ,, | 0·1 (acetone only) | — | ,, | 10 | 5 | Papillomata appeared in 2d much later than in 2a. Results of painting confirmed earlier observations (Allsopp, 1946). (Fig. 1.) |
| 3a | 10 | 20 ,, | Benzpyrene in acetone | 0·1 | 0·05 | 3–4 days alternately with croton oil | 0·1 | 0·5 | 3–4 days alternately with benzpyrene | 8 | 8 | Appearance of papillomata accelerated by croton oil. (Fig. 1.) |
| b | 9 | 20 ,, | Ditto | 0·1 | 0·05 | Ditto | 0·1 (acetone only) | — | Ditto | 8 | 4 | Experiment terminated at 20 weeks as oil appeared to have general toxic effect. |

hair brush into an area about 1 cm. in diameter on the back of each mouse. The fur was not clipped. Solutions of croton oil and of 3:4-benzpyrene in acetone were applied by pipette drop by drop on a similar area of unclipped fur and were allowed to dry.

*Histology.*—The histological material was fixed in acetic Zenker's fixative. Serial sections were cut at 10$\mu$ and stained with haematoxylin and eosin.

### RESULTS.

The number of mice used, the treatment, and the general nature of the results of each experiment, are summarized in Table I, and the times of appearance of growths are shown diagrammatically in Fig. 1.

FIG. 1.—Times of appearance of papillomata and epitheliomata on mice painted with croton oil and water-soluble materials derived from 3:4-benzpyrene, Experiment 2, and with croton oil and 3:4-benzpyrene itself, Experiment 3.

Experiment 2a : - - - - - Papillomata subsequently sloughed. ——— Papillomata which developed into epitheliomata.

Experiment 2d : Short lines indicate epitheliomata observed in earlier experiments (Allsopp, 1946).

### *Changes produced in mouse-skin by painting with croton oil only.* (*Table I, Experiment* 1.)

A single painting with 0·1 ml. of 0·5 per cent croton oil in acetone (Experiment 1a) at first caused little superficial change. Slight ulceration, as evidenced by patchy encrustation, was visible on the third day, after which epilation began. This was complete by about the tenth day. So soon as the encrustation sloughed hair began to grow again, and by the fourteenth day the animals appeared normal. Day to day observations are set out in Table II.

Painting with 0·5 per cent croton oil at weekly intervals (Experiment 1b) caused a similar sequence of macroscopic changes, but restoration of hair growth was delayed until the third week, after which the epilation cycle repeated itself

TABLE II.—*Effect of Single Application of* 0·1 *ml. of* 0·5 *per cent Croton Oil in Acetone.*

| Day after painting. | Appearance. Superficial. | Histological. |
|---|---|---|
| 1 | No obvious change | Diffuse leucocytic infiltration of the dermis and hyperplasia of epidermis. |
| 2 | Very slight ulceration | Ditto. |
| 3 | Encrustation beginning | The same with ulceration and epilation. |
| 4 | Skin lightly encrusted | Less hyperplasia and less infiltration. |
| 5 | Epilation beginning | Hyperplasia and infiltration. Follicular regeneration beginning. |
| 6 | Epilation and encrustation marked | The same, with ulceration. |
| 7 | Extensive epilation | Ditto. New deep dermal connective tissue. |
| 9 | Epilation complete | Ditto. New subdermal connective tissue. |
| 11 | Hair growing | The same, but only in minute patches. |
| 14 | Hair re-growing | No abnormality. |

at approximately two-week intervals. Croton oil 1 per cent (Experiment 1c) behaved similarly, but gave rise to much more severe ulceration. Applied to the skin in this way, croton oil seemed to have a general toxic effect on the mice, and it was found inadvisable to continue paintings for more than twenty weeks.

*Co-carcinogenic effect of croton oil with a bicarbonate extract from irradiated* 3:4-*benzpyrene.* (*Table I*, *Experiment* 2.)

Experiments 2a and 2d: In Experiment 2a the mice were painted with bicarbonate extract from irradiated benzpyrene 4 to 5 times weekly and with croton oil in acetone once weekly until the growths produced became malignant. In Experiment 2d, which repeated the earlier tests of the bicarbonate extracts (Allsopp, 1946), acetone alone was substituted for the croton oil solution. Papilomata appeared in Experiment 2a after 13 weeks. The first was observed in 2d in the thirty-second week (Fig. 1), by which time 9 of the 20 mice in 2a had developed 10 papillomata between them. Those on 7 of these 9 mice, however, were observed to keratinize and slough; only 3 of these early papillomata, 2 of which were on the same animal, continued to grow until they developed into carcinomata, this stage being reached about the thirty-ninth week. Three more mice developed papillomata between the thirtieth and thirty-seventh weeks: two of these growths became malignant, and one (the last to appear) sloughed. Epitheliomata also appeared during this period on 2 mice which had previously lost papillomata by sloughing elsewhere on the painted area. (These are not recorded in Fig. 1.) Subsequent histological examination of all the sites at which sloughing had occurred revealed hyperplasia; in 2 cases there was also hyperkeratosis. There were signs of ulceration on 2 animals, but it was very slight (Experiment 1). There were no signs of the papillomata.

In Experiment 2d papillomata appeared on 5 out of 10 mice between the thirty-second and seventieth weeks of painting. None sloughed, and they subsequently developed into squamous cell carcinomata. These results confirm those of the earlier tests (Allsopp, 1946 ; Fig. 1) in which carcinomata developed on 7 out of 10 mice between the thirty-fifth and sixty-sixth weeks of painting. These earlier results are included for comparison in Fig. 1, Experiment 2d.

Control Experiments 2b and 2c : One set of 10 mice (2b) was painted daily with a bicarbonate extract from unirradiated benzpyrene (" Control extract " in Table I), and another (2c) with bicarbonate, each set also receiving a weekly application of croton oil. These experiments were continued for 39 weeks, by which time 13 growths had been recorded on the 20 mice in Experiment 2a. No growths resulted, and histological examination of the painted skins revealed nothing more than a slight hyperplasia on any animal.

*Co-carcinogenic effect of croton oil with* 3:4-*benzpyrene.* (*Table I, Experiment* 3.)

Equal volumes (0·1 ml.) of 0·05 per cent benzpyrene in acetone and of 0·5 per cent croton oil in acetone were applied alternately at 3- to 4-day intervals, each animal thus receiving each agent once weekly. Control animals were painted at the same times with benzpyrene, but with acetone instead of croton oil solution. Papillomata were first seen on the experimental animals after 5 weeks ; all the survivors had malignant or " pre-cancerous " lesions by the fourteenth week, a precancerous lesion in this context being one exhibiting irregular epilation, hyperkeratosis, abnormal regeneration of hair follicles, ill-defined epidermal projections, a large increase in the number of mitotic cells, numerous abnormal mitotic figures, and leucocytic infiltration of the dermis (Allsopp, 1946). The first papilloma appeared on a control animal in the sixteenth week. When the experiment was discontinued in the twentieth week, 4 out of 8 surviving control animals had cancerous or pre-cancerous lesions ; the remainder showed marked hyperplasia only. The croton oil had thus caused an acceleration of the production of papillomata (Fig. 1, Experiments 3a and 3b).

### DISCUSSION.

When the action of a carcinogen is tested in combination with some other external physical or chemical agent, this agent has been described as " co-carcinogenic " when the added treatment causes a higher yield of tumours or a shortening of the latent period before they appear (Berenblum, 1947). On the basis of extensive experimental work by Berenblum (1944), substantiated by subsequent workers, croton oil has become recognized as a most potent co-carcinogen. The experiments described above, however, suggest that its action is more complicated than this simple description migth indicate.

Despite a marked difference in the response to croton oil of the mice now used when compared with that of strains used by other workers, Berenblum's findings (Berenblum, 1944) have been largely confirmed when 3:4-benzpyrene was the carcinogen ; but when the hydrocarbon was replaced by the water-soluble substances which are obtained from it by the action of ultraviolet light croton oil appeared to exert a different effect since, while papillomata appeared on the mice at a very much earlier stage than they did with the carcinogenic solutions alone, a proportion of these papillomata did not develop into epitheliomata, whereas painting with the water-soluble derivatives of benzpyrene by themselves produced carcinomata. The distinction in behaviour, however, was not entirely clear-cut, since the papillomata on 2 out of 9 animals did persist, growing very slowly, until they became malignant.

The earlier experiments with the water-soluble substances (Allsopp, 1946) showed that with them the process of carcinogenesis was slow, but that it passed through the usual 3 phases of hyperplasia, papillomata, and finally carcinomata. The last stage required 6 to 10 weeks. The present experiments suggest that croton oil accelerates the transition from hyperplasia to papillomata—the first papilloma appeared after 13 instead of 26 weeks—but not the transition from papillomata to epitheliomata, which again took about 9 weeks. Indeed it may even delay this change, or—in those cases where the papillomata were sloughed—prevent it. In contrast to this, benzpyrene itself appears to act rather more quickly ; epitheliomata had developed in the animals recorded in Experiment 3b of Fig. 1 between the first appearance of papillomata at the sixteenth and eighteenth weeks and the conclusion of the experiment in the twentieth week.

It might appear possible that the sloughing of the early papillomata on the animals painted with croton oil and the bicarbonate extracts from irradiated benzpyrene could be attributed, at least in part, to ulceration caused by croton oil ; but the ulceration produced by croton oil at the concentration which was used in the experiments was very slight (Experiment 1), and no sloughing was observed when the same concentration of croton oil was used in conjunction with 3:4-benzpyrene. This, therefore, is not the main factor in the sloughing process. Sloughing of early papillomata has, however, been observed in experiments in which mice were painted with cholanthrene and croton oil (Allsopp, 1948), while Shubik (1950) has reported a diminished number of malignant tumours, and a very high regression rate, in mice painted with croton oil in conjunction with 9:10-dimethyl-1:2-benzanthracene. It thus appears that while croton oil is co-carcinogenic in the sense that it causes papillomata to appear on mice treated with a carcinogen at an earlier stage than they would with the carcinogen alone, it does not always convert these papillomata into epitheliomata, and that the co-carcinogenic effect is influenced considerably by the carcinogen used.

### SUMMARY.

When water-soluble materials derived from 3:4-benzpyrene were painted on the skins of mice in conjunction with croton oil as co-carcinogen, papillomata appeared much earlier than with the water-soluble materials alone ; but most of these *early* papillomata keratinized and sloughed, whereas those produced by the water-soluble materials alone grew into epitheliomata.

These experiments were begun at the Strangeways Research Laboratory, Cambridge. I am indebted to Dr. A. Glucksmann, of that laboratory, for examining and reporting on all the histological material, and for much valuable advice and discussion ; and to Mr. C. Winter, his assistant, for preparing that material. I am grateful to Dr. P. Gorer, of Guy's Hospital Medical School, and to Dr. M. Salaman, of the London Hospital, for comparing with mine the results of their experiments on the effects of croton oil on the skin of mice. The work described was supported by the British Empire Cancer Campaign, for whose assistance I express grateful thanks. A preliminary account appeared in the 25th Annual Report of the Campaign (Allsopp, 1947).

### REFERENCES.

ALLSOPP, C. B.—(1946) *Cancer Res.*, **6**, 24.—(1947) *Ann. Rep. Brit. Emp. Cancer Campgn.*, **25**, 165.—(1948) *Ibid.*, **26**, 240.

*Idem* AND SZIGETI, B.—(1946) *Cancer Res.*, **6**, 14.

BERENBLUM, I.—(1941) *Ibid.*, **1**, 44, 807.—(1944) *Arch. Path.*, **38**, 233.—(1947) *Brit. med. Bull.*, **4**, 343.

SHUBIK, P.—(1950) *Cancer Res.*, **10**, 713.